# The body weight-walking distance product as a superior parameter in determining the VO_2_ on-kinetics in coronary artery disease

**DOI:** 10.1590/1414-431X2025e14367

**Published:** 2025-05-30

**Authors:** I.S. Rocco, W.J. Gomes, M. Viceconte, B.C. Matos-Garcia, F.S. Menezes-Rodrigues, F.S. Tallo, R.M. Arida, N.A. Hossne, R. Arena, S. Guizilini

**Affiliations:** 1 Programa de Pós Graduação em Cardiologia e Disciplina de Cirurgia Cardiovascular, Universidade Federal de São Paulo, São Paulo, SP, Brasil; 2 Departamento de Ciências do Movimento Humano, Escola de Fisioterapia, Universidade Federal de São Paulo, Santos, SP, Brasil; 3 Programa de Pós-Graduação em Ciência Cirúrgica Interdisciplinar, Escola Paulista de Medicina, Universidade Federal de São Paulo, São Paulo, SP, Brasil; 4 Disciplina de Medicina Clínica, Escola Paulista de Medicina, Universidade Federal de São Paulo, São Paulo, SP, Brasil; 5 Departamento de Fisiologia, Universidade Federal de São Paulo, São Paulo, SP, Brasil; 6 Department of Physical Therapy and Integrative Physiology Laboratory, College of Applied Health Sciences, University of Illinois Chicago, Chicago, IL, USA

**Keywords:** Exercise capacity, Coronary heart disease, Oxygen uptake, Six-minute walk test, Cardiorespiratory fitness

## Abstract

The 6-minute walk test is frequently used to assess the functional capacity of the cardiac disease population. Nevertheless, anthropometric differences can confound or misestimate performance, which highlights the need for new parameters. This study aimed to investigate the potential of the body weight-walking distance product (D·W) compared to the 6-minute walk test distance to predict exercise capacity measured by oxygen uptake (VO_2_) on-kinetics in coronary artery disease (CAD) patients. A cross-sectional study was conducted in a tertiary-care reference institution. Forty-six participants with multivessel CAD with and without left ventricular dysfunction underwent a 6-minute walk test with simultaneous use of mobile telemetric cardiopulmonary monitoring to evaluate VO_2_ kinetics and other cardiorespiratory responses. The Borg rating of perceived exertion for lower limb discomfort was only correlated with the D·W (P=0.007). The percent predicted and actual distance were only modestly to moderately correlated with VO_2_ on-kinetics (R^2^=0.12 and R^2^=0.29, P<0.05). All the associations of VO_2_ on-kinetics parameters were improved, showing a stronger correlation to the D·W (R^2^=0.49, P<0.0001), which also had a larger effect size to identify differences between coronary disease patients compared to distance walked (*d*=1.32 *vs d*=0.84). The D·W demonstrated potential to be better than the distance walked in determining VO_2_ on-kinetics in participants with CAD with and without left ventricular dysfunction.

## Introduction

The gold standard method to assess exercise capacity is the cardiopulmonary exercise testing, in which the peak oxygen uptake (VO_2peak_) is measured under maximal effort (1). However, activities of daily living mostly require a submaximal level of exertion. Submaximal exercise testing performed with a constant load allows the evaluation of the oxygen uptake (VO_2_) transition from rest to exercise, defined as VO_2_ on-kinetics (2,3). As with VO_2peak_, VO_2_ on-kinetics has been shown to be an important prognostic marker in several chronic diseases (4), particularly in coronary artery disease (CAD) with left ventricular systolic dysfunction (LVSD). In clinical practice, a number of field tests based on walking performance are frequently performed to assess submaximal functional capacity in these patients (5).

The 6-minute walk test (6MWT) is easily administered and represents the effort of daily-life activities (6). In a fashion similar to the constant load ergometer testing, simultaneous use of mobile telemetric cardiopulmonary monitoring (MOB) while performing the 6MWT can provide the assessment of the VO_2_ on-kinetics (7). The main parameter of the 6MWT is the distance walked in meters (8). However, the measurement of walking distance is limited in accurately predicting exercise capacity due to the presence of confounding factors (i.e., body weight, step length) (9) and is not clinically useful for less impaired patients (5,7,8).

Chuang et al. (10) have described a measurement of walking work, similar to the work on a treadmill, represented as the product of the body weight and distance (D·W), which may improve the evaluation capacity of the 6MWT beyond distance. The D·W has been reported to better correlate with VO_2peak_ and VO_2_ at ventilatory threshold compared to walking distance in patients with chronic obstructive pulmonary disease (10,11). Nevertheless, to our knowledge, there is no current analysis of whether the D·W could be more effective than the 6MWT distance in assessing submaximal exercise capacity in patients with CAD without a severe impairment. A stronger correlation between the D·W product and VO_2_ on-kinetics could help enhance clinical utility of the 6MWT by providing a more sensitive and specific measure of a patient"s submaximal exercise capacity, with potentially greater analytical power and effect size compared to the 6MWT. Thus, the aim of the present study was to investigate the potential of the D·W, compared to 6MWT distance, to reflect variations in VO_2_ on-kinetics in CAD patients with and without LVSD.

## Material and Methods

### Participants and study design

This cross-sectional study was conducted at the academic Hospital of Universidade Federal de São Paulo and patients were recruited from the myocardial disease clinic. Participants with a confirmed diagnosis of stable CAD were included in this study, which was approved by the Institutional Ethics Committee (number 1.424.088), in accordance with the ethics code of the Declaration of Helsinki. All participants were informed about the study and signed a written consent form.

CAD diagnosis was obtained by coronary angiography. Through echocardiography, a left ventricular ejection fraction (LVEF) <45% was used as the threshold to define participants with LVSD (12). Exclusion criteria consisted of recent myocardial infarction (<6 months), presence of chronic or acute pulmonary disease, morbid obesity, neurological or orthopedic diagnoses affecting the ability to complete the study protocol, inability to comprehend and perform the tests, or hemodynamic instability/severe arrhythmias during test protocols.

### 6-minute walk test

Submaximal functional capacity was evaluated using the 6MWT, according to the American Thoracic Society (ATS) recommendations (13). Prior to the 6MWT, volunteers rested on a chair for 3-5 min to record baseline parameters. At the end of the 6MWT, participants rested in a seated position for the recovery phase. Two physical therapists with expertise in the assessment conducted the field test in the morning in a 30-m indoor flat hallway in the clinic.

Dyspnea and leg discomfort were rated using the Borg rating of perceived exertion (RPE) scale before, after, and every 2 min during the 6MWT. The total distance covered during the 6-minute test was recorded in meters. D·W was calculated by multiplying the distance in kilometers (km) and body weight in kilograms (kg) (10). The prediction equation proposed by Soares and Pereira (14) was used to predict the walking distance of all participants. Two tests were performed in a single visit with a 1-h rest between the tests to assess the learning effects of the test and to familiarize patients with the equipment. The test was interrupted based on ATS criteria (13): intolerable dyspnea (RPE>7), angina or chest pain or tightness, dizziness, pallor, vertigo, palpitations, leg cramps, or severe lower limb discomfort (RPE>7), oxygen desaturation (SpO_2_<9%), abnormal gait patterns, and gait balance alterations.

### MOB device during 6MWT

The 6MWT was performed with a simultaneous mobile telemetric cardiopulmonary monitoring (MOB) device (Oxycon Mobile-Viasys Healthcare, USA) to measure real time breath-by-breath cardiopulmonary responses (7). The unit was harnessed to the participants in a way that their walking was not affected. Heart rate was recorded by a 12-lead electrocardiogram (Oxycon ECG module, Viasys Healthcare). A facemask (with a dead space <70 mL) linked to a turbine volume transducer was used to continuously sample gas exchange, tidal volumes, and breathing frequency. Breath-by-breath calculations of VO_2_ and carbon dioxide output (VCO_2_) were then digitized. Before each 6MWT, spirometry was performed with the MOB device according to ATS guidelines (15) for standardization of volume measures. Steady-state variables were calculated as an average of the last 2 min of exercise. The transition of VO_2_ through exercise was registered to obtain the curve fitting of VO_2_ on-kinetics.

### Curve fitting of VO_2_ kinetics

Raw breath-by-breath data obtained from the MOB device were preprocessed by the average of consecutive 15-s periods. The fit of VO_2_ on-kinetics was performed by a monoexponential regression model (2,16), as follows:

$$\text{f(t)}=\text{y}0+(\text{y}1-\text{y}0)(1-{\text{e}}^{-\text{t/T}})$$ (Eq. 1)

where the f(t) represents VO_2_ at a certain time (t); y0 indicates the lower limit at t=0, i.e., the VO_2_ at rest (the mean value of the last minute VO_2_ prior to the test); y1 represents the upper limit, indicating the steady-state VO_2_ (VO_2SS_); and τ indicates the time constant, i.e., the time needed to reach 63% of the VO_2SS_. Since time delay was found to be undistinguishable from the second exponential phase (17), phase I was not modelled in this study. Since the time delay was not taken into account, the time needed for a 63% increase in VO_2SS_, i.e., time constant (τ), also corresponds to the mean response time (MRT) (17). The MRT was corrected for the work rate (wMRT) during the 6MWT to avoid possible differences in intensity amongst participants. The work rate was obtained using the difference between VO_2_ at rest and VO_2_ during effort (VO_2SS_-VO_2rest_) (18,19). The quality of fit was assessed visually by two independent investigators to avoid data with obvious lack-of-fit, leading to 6 exclusions.

### Statistical analysis

Categorical data are reported as absolute and relative frequency and continuous variables are reported as means±SD. The Shapiro-Wilk test was used to investigate the normality distribution of data. Pearson’s correlation test was performed to investigate the relationship between VO_2_ on-kinetics and 6MWT parameters to extract coefficients and compare the performance of D·W, distance, and predicted distance. Comparison of 6MWT performance in CAD participants with and without LVSD was assessed using the unpaired Student’s *t*-test or Mann-Whitney U-test, according to data distribution. Cohen’s *d* test was used to investigate the effect size of 6MWT parameters (20). A *post hoc* sample size analysis was performed to determine whether the effect size of the 6MWT parameters would evolve with satisfying power of analysis. The statistical analyses were performed using Jamovi (2.3.21.0) and R Studio (2023.03.1). A P value <0.05 was considered statistically significant for all tests.

## Results

**Figure 1:**
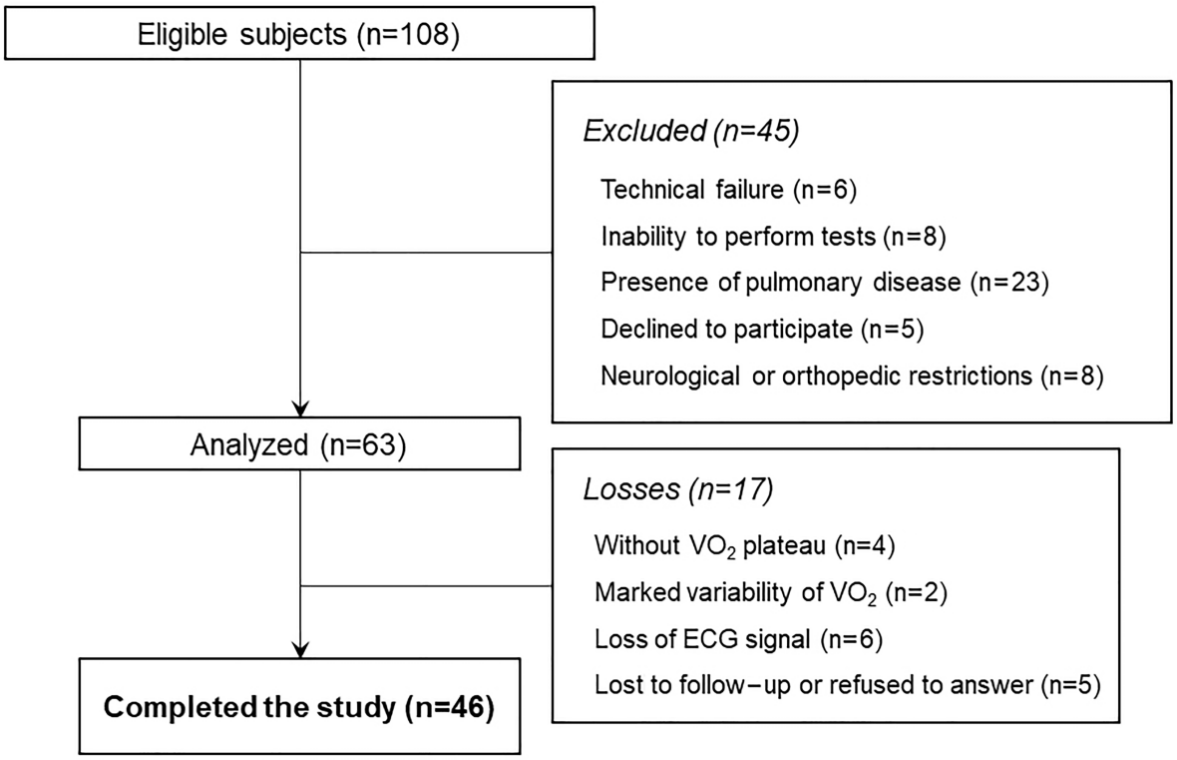
Flow-chart of the study evaluation protocol.

The characteristics of the final 46 volunteers (Figure 1) are presented in Table 1. The VO_2_ oxygen on-kinetics was successfully fitted, achieving an average of 3.8±0.9 metabolic equivalents (METS) during the 6MWT. The mean VO_2SS_ was 909.8±23.7 mL/min, τ was 53.3±9.12 s, and wMRT was 1.64±1.0×10^-3^ min^2^/mL. All physiological responses to the 6MWT significantly changed at steady state compared with rest, except for VE/VO_2_ (Table S1).

**Table 1:** Demographic and clinical characteristics of volunteers.

Variable	n=46
Age (years), mean (SD)	60.2 (7.6)
Sex (M/F)	37/9
Weight (kg), mean (SD)	70.06 (10.45)
BMI (kg/m^2^), mean (SD)	26.25 (8.26)
Hypertension (%)	82.3
Diabetes (%)	32.3
LVEF, mean (SD)	0.51 (0.15)
Main affected artery (%)	
LAD	46.35
RCA	39.65
Cx	1.05
Others	12.95
6MWT (m), mean (SD)	441 (67)
%Predicted-distance, mean (SD)	82.1 (11.3)
D·W (km/kg), mean (SD)	30.9 (67.9)

BMI: body mass index; LVEF: left ventricular ejection fraction; LAD: left anterior descending artery; RCA: right coronary artery; Cx: circumflex artery; 6MWT: 6-minute walk test; D·W: body weight-walking distance product.

VCO_2_, METS, VE, change in VO_2_, and RPE for dyspnea were all significantly and positively-correlated to the 6MWT parameters (Table 2). However, all associations were stronger when D·W was used. Notably, the RPE for lower limb discomfort was only correlated with D·W (Table 2).

**Table 2:** Association between 6-minute walk test parameters and submaximal exercise performance.

	Distance (m)			% predicted			D·W	
	r	P value		r	P value		r	P value
ΔVO_2_ (mL/min)	0.61	<0.001		0.36	0.034		0.74	<0.001
VCO_2_ (mL/min)	0.63	<0.001		0.37	0.029		0.79	<0.001
RER	0.13	0.439		-0.008	0.960		0.21	0.239
METS	0.67	<0.001		0.48	0.004		0.69	<0.001
VE (L/min)	0.54	0.001		0.29	0.089		0.69	<0.001
VE/VCO_2_	-0.28	0.101		-0.23	0.189		-0.22	0.210
BR (%)	-0.10	0.567		0.05	0.749		-0.27	0.140
ΔRPE, dyspnea	0.38	0.030		0.35	0.045		0.44	0.012
ΔRPE, limb discomfort	0.21	0.229		0.17	0.348		0.45	0.007

Pearson’s correlation test was used to investigate the associations. D·W: body weight-walking distance product; Δ: change from rest to the steady-state; VO_2_, oxygen uptake; VCO_2_: carbon dioxide output; RER: respiratory exchange ratio; METS: metabolic equivalents; VE: ventilatory equivalent; VE/VCO_2_: ventilatory equivalent of carbon dioxide; BR: breathing reserve; RPE: Borg rating of perceived exertion scale.

**Figure 2:**
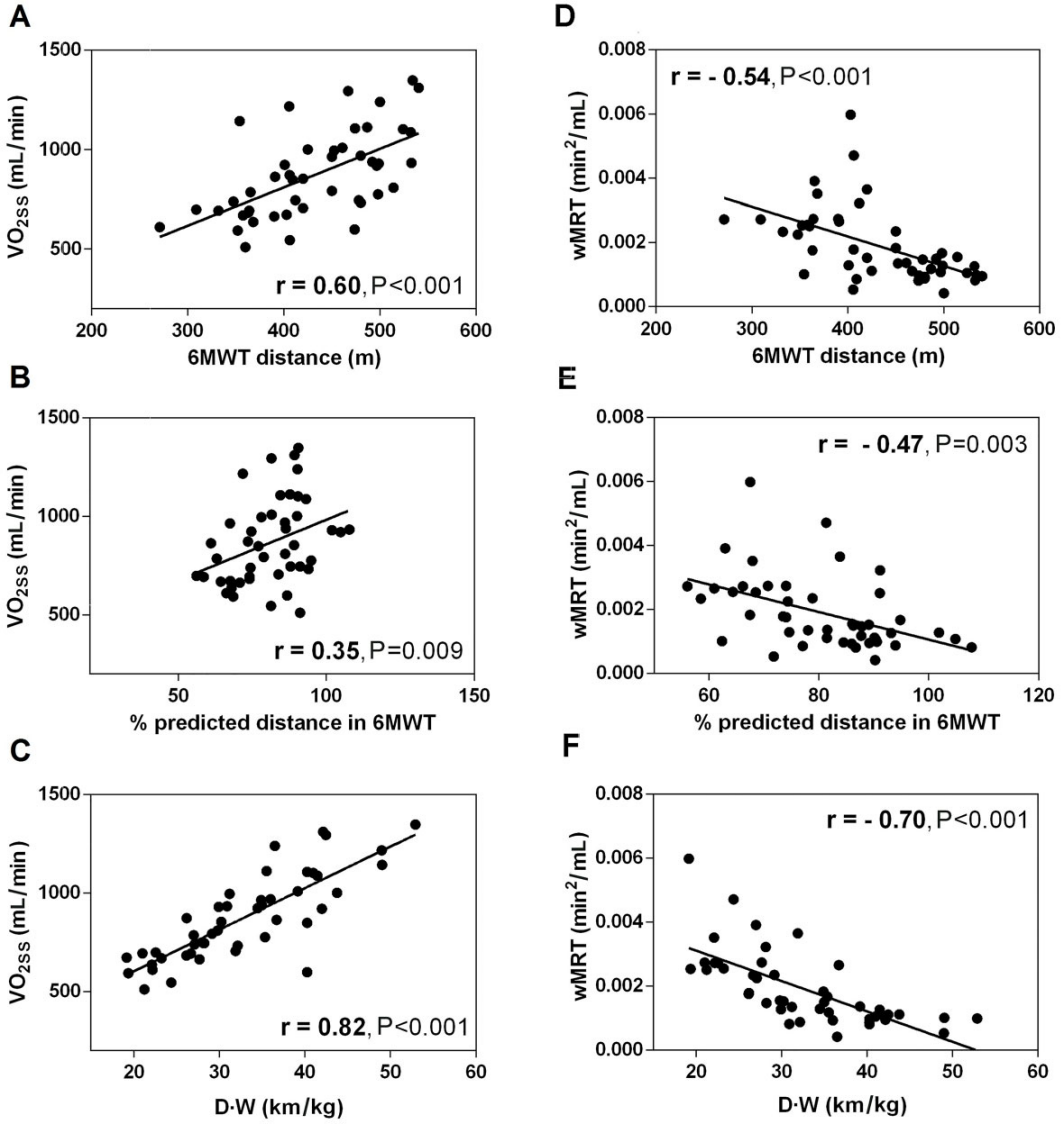
Pearson's correlation test to investigate association of steady-state oxygen uptake (VO_2SS_) and work rate mean response time (wMRT) in relation to 6-minute walk test (6MWT) parameters. D·W: body weight-walking distance product.

The 6MWT distance was positively and moderately correlated with VO_2SS_ (R^2^=0.36, P<0.0001; Figure 2A). The 6MWT percent predicted distance was only positively and modestly correlated with VO_2SS_ (R^2^=0.12, P=0.009; Figure 2B), while the D·W was strongly correlated with VO_2SS_ (R^2^=0.67, P<0.001; Figure 2C).

The 6MWT distance had a negative and moderate association with wMRT (R^2^=0.29, P<0.001; Figure 2D). The correlation between 6MWT percent predicted distance and the wMRT was negatively and moderately correlated (R^2^=0.22, P=0.003; Figure 2E), whereas the wMRT association was improved by showing a negatively and strong correlation with D·W (R^2^=0.49, P<0.001; Figure 2F).

There was a stronger positive correlation between D·W and LVEF compared with the walking distance or the % predicted distance (r=0.72, P<0.001 *vs* r=0.55, P=0.001 or r=0.43, P=0.005, respectively). When participants were dichotomized according to LVEF, those with LVSD (LVEF<45%) had worse submaximal functional capacity compared to participants without LVSD (LVEF >45%). Participants with LVSD achieved a lower 6MWT distance and lower D·W (Table 3). The effect size Cohen’s *d* test demonstrated that D·W was superior in identifying the difference between the groups compared with the 6MWT distance and percent predicted distance (*d*=1.32 *vs d*=0.84 and *d*=0.02, respectively; Table 3). Also, because a large effect size was found when D·W was used, the analysis of group comparisons achieved a power of 95% (Table 3).

**Table 3:** 6MWT parameters to differentiate subjects according to absence or presence of LVSD.

	LVEF≥45%(n=24)	LVEF<45%(n=22)	Power	Effect size^&^	*d* lower limit	*d* upper limit
Distance (m)	463.7 (62.9)	412.3 (62.6)*	0.51	0.84	-24.32	27.01
% Predicted	82.2 (9.1)	81.9 (13.9)*	0.01	0.02	-3.64	5.84
D·W (km/kg)	34.26 (6.25)	27.05 (4.77)*	0.96	1.32	-1.19	3.31

Data are reported as mean (standard deviation). D·W: body weight-walking distance; LVEF: left ventricular ejection fraction; 6MWT: 6-minute walk test. *P<0.01 comparison between groups. ^&^Cohen’s *d* effect size.

## Discussion

The current study demonstrated that D·W had a better relationship with VO_2_ on-kinetics than 6MWT distance and percent predicted distance in patients with CAD. Moreover, D·W had a larger effect size than the 6MWT distance in CAD patients with and without LVSD. These findings support the premise that D·W is an easily applicable and potentially meaningful measure of submaximal exercise performance and physiologic health during exertion, even in patients with less severe impairment.

Field tests have been used to assess exercise capacity in people with chronic diseases for many years. Submaximal exertion tests enable greater patient toleration and provide a stronger indication of the ability to perform daily activities (5,8,13). In this context, the 6MWT has been used as a prognostic marker for several diseases, such as cardiac disease, particularly LVSD (6,21,22). Nevertheless, the 6MWT distance alone can only detect severe exercise limitation, i.e., patients unable to walk more than 300 m. To acquire physiological insights, several research groups around the world have simultaneously employed a MOB device during the 6MWT (18,19,23). The breath-by-breath analysis of cardiopulmonary responses more accurately quantifies physiologic health and the degree of exercise limitation. Furthermore, the analysis of VO_2_ during the initial phase of the 6MWT allows for the assessment of VO_2_ on-kinetics, as in our study, which is linked to the risk of future adverse events (19,24).

The walking distance (in meters) has been historically recognized as the primary variable of the 6MWT. Several studies have reported threshold 6MWT distance values to predict increased risks of adverse events, such as myocardial infarction, stroke, re-hospitalization, and mortality (25). Despite its wide use, studies have reported only a moderate association between the 6MWT distance and VO_2peak_ and other cardiopulmonary exercise testing parameters (8). In fact, we also found a moderate relationship between 6MWT distance with VO_2SS_ and wMRT in our CAD patients, explaining the discrepancy from the previously known relationship between METS and walking speed. This finding suggests that the use of the 6MWT distance can only predict obvious impairment of functional capacity and therefore an already predictable risk of morbidity and mortality.

Additionally, predicted values based on age, gender, and body mass index have been studied for populations around the world to obtain normative performance values. Since reference values are established for each country separately, they are difficult to apply as a global parameter. Moreover, in the current study, only a modest association was found with VO_2_ on-kinetics when the percent predicted values of the 6MWT were used. The lack of a stronger association could indicate that the functional capacity of the participant during exercise is not well represented by the current prediction models.

The current limitations of the physiological information derived from the 6MWT encourage the search for new parameters that more accurately reflect functional performance (9) and overall health status. Previous reports have already indicated that the work of walking during the 6MWT can be correlated with the horizontal work on a treadmill (W_HO_). Since the 6MWT is performed on a horizontal surface and at constant velocity, the work of walking in the 6MWT can be calculated as a product of distance and weight (i.e., D·W) (10). In our study, we found that all associations with cardiorespiratory responses were greatly improved when D·W was used rather than walking and percent predicted distance values. We observed a moderate-to-modest correlation between both VO_2SS_ and wMRT with walking and percent predicted distance. However, when D·W was applied, a strong correlation was observed between VO_2SS_ (r=0.82, P<0.001) and wMRT (r=-0.70, P<0.001). These results corroborate other findings in the literature. Chuang et al. (10) found a modest correlation between 6MWT distance and VO_2peak_ (r=0.40, P<0.05) *vs* a stronger correlation between D·W and VO_2peak_ (r=0.67, P<0.05). Similarly, Poersch et al. (11) found that VO_2peak_ was modestly correlated with distance (r=0.32, P=0.084) and percent predicted distance using Soares and Pereira (14) equation (r=0.35, P=0.058), while strongly correlated with D·W (r=0.76, P<0.01). Both aforementioned studies were performed in chronic obstructive pulmonary disease cohorts that underwent cardiopulmonary exercise testing. To the best of our knowledge, this is the first study to evaluate the association between D·W of the 6MWT with VO_2_ on-kinetics in CAD patients.

The present investigation revealed a markedly greater association between wMRT and D·W. The wMRT indicates the duration required to reach the steady-state phase during constant load exercise. The dynamics of oxygen uptake at the onset of exercise serve as a reliable indicator of the body’s capacity to mobilize physiological reserves in response to exercise demands. Kern et al. (19) observed that higher wMRT correlated with increased mortality rates in cardiac patients, indicating that slowed oxygen kinetics may reflect broader cardiovascular and metabolic dysfunction. Moreover, Rocco et al. (24) recognized slower wMRT as an indicator of early postoperative complications and poorer outcomes in cardiac surgery patients, highlighting its prognostic significance. The published findings indicate that wMRT serves as a vital measure of functional ability and a potential prognostic predictor. According to the referenced literature, faster wMRT may be associated with enhanced survival rates and extended lifespans, presumably because of greater circulatory efficiency and increased muscular oxygen utilization. By enhancing the statistical coefficient in correlation analyses, D·W emerged as a variable that connects physiological performance and may possess long-term prognostic significance that warrants further investigation.

It is already well established that CAD exposes the myocardium to a mismatch in oxygen supply and demand. The longer this imbalance persists, the more severe the CAD becomes, leading to a greater impairment in ventricular function, as in LVSD. CAD is the major cause of chronic heart failure, leading to progressive impairment in exercise capacity (26). A limitation of physical activity in this population can be detected by a lower 6MWT performance (6). In the current study, LVEF was moderately to strongly correlated with all three 6MWT parameters evaluated in our CAD cohort. As expected, based on previous literature (22), our results also revealed that when participants were separated according to LVEF, those with LVSD achieved lower distance and lower D·W during the 6MWT.

Although the 6MWT distance already had a large effect size (*d*=0.84), the D·W effect size was much larger (*d*=1.32), indicating that D·W is a powerful parameter to discriminate the level of disease impairment. These findings suggest that D·W may be a preferred measure for quantifying submaximal performance. Moreover, the stronger correlation observed with VO_2_ on-kinetics supports the widespread use of D·W, particularly if ventilatory expired gas analysis is not available.

A limitation of this study was that most participants were males (80.4%). Women have a lower exercise capacity, and as such, the results may be affected by a gender bias, especially regarding the utilization of a walking test (27-29). This was a cross-sectional study with a modest number of patients. Future research is necessary to establish specific D·W values that could predict clinical outcomes in cardiac patients and confirm this parameter as being superior to walking distance.

### Future directions

The 6MWT as an assessment of functional capacity needs to be modified to include other variables besides walking distance in order to detect more refined responses to submaximal exertion. The oxygen uptake on-kinetics during the 6MWT has been investigated as a predictive marker for cardiac patients, but it requires specialized equipment to be carried out. Although the D·W product has a predictive relationship with VO_2_ on-kinetics, more research is needed to determine which responses are relevant to prognosis in cardiac patients.

## Conclusions

The D·W is a potentially superior measure than the 6MWT distance in determining VO_2_ on-kinetics in participants with CAD. D·W seems to reflect the work performed by walking and may be a stronger parameter for the evaluation of submaximal exercise capacity and performance, especially if a cardiopulmonary testing device is not available.

## Supplementary Material

Click here to view [pdf].

## References

[1] Balady GJ, Arena R, Sietsema K, Myers J, Coke L, Fletcher GF (2010). Clinician’s guide to cardiopulmonary exercise testing in adults:
a scientific statement from the American Heart Association. Circulation.

[2] Whipp BJ, Wasserman K (1972). Oxygen uptake kinetics for various intensities of constant-load
work. J Appl Physiol.

[3] Xu F, Rhodes EC (1999). Oxygen uptake kinetics during exercise. Sports Med.

[4] Schalcher C, Rickli H, Brehm M, Weilenmann D, Oechslin E, Kiowski W (2003). Prolonged oxygen uptake kinetics during low-intensity exercise
are related to poor prognosis in patients with mild-to-moderate congestive
heart failure. Chest.

[5] Casillas JM, Hannequin A, Besson D, Benaïm S, Krawcow C, Laurent Y (2013). Walking tests during the exercise training: specific use for the
cardiac rehabilitation. Ann Phys Rehabil Med.

[6] Bittner V, Weiner DH, Yusuf S, Rogers WJ, McIntyre KM, Bangdiwala SI, SOLVD Investigators (1993). Prediction of mortality and morbidity with a 6-minute walk test
in patients with left ventricular dysfunction. JAMA.

[7] Tueller C, Kern L, Azzola A, Baty F, Condrau S, Wiegand J (2010). Six-minute walk test enhanced by mobile telemetric
cardiopulmonary monitoring. Respiration.

[8] Guazzi M, Dickstein K, Vicenzi M, Arena R (2009). Six-minute walk test and cardiopulmonary exercise testing in
patients with chronic heart failure: a comparative analysis on clinical and
prognostic insights. Circ Heart Fail.

[9] Pepera GK, Sandercock GR, Sloan R, Cleland JJF, Ingle L, Clark AL (2012). Influence of step length on 6-minute walk test performance in
patients with chronic heart failure. Physiotherapy.

[10] Chuang ML, Lin IF, Wasserman K (2001). The body weight-walking distance product as related to lung
function, anaerobic threshold and peak VO_2_ in COPD
patients. Respir Med.

[11] Poersch K, Berton DC, Canterle DB, Castilho J, Lopes AL, Martins J (2013). Six-minute walk distance and work relationship with incremental
treadmill cardiopulmonary exercise test in COPD. Clin Respir J.

[12] Zile MR, Brutsaert DL (2002). New concepts in diastolic dysfunction and diastolic heart
failure: part I: diagnosis, prognosis, and measurements of diastolic
function. Circulation.

[13] ATS Committee on Proficiency Standards for Clinical Pulmonary
Function Laboratories (2002). ATS Statement: Guideline for the six-minute walk
test. Am J Respir Crit Care Med.

[14] Soares MR, Pereira CA (2011). Six-minute walk test: reference values for healthy adults in
Brazil. J Bras Pneumol.

[15] American Thoracic Society (1995). Standardization of spirometry. 1994 Update. Am J Respir Crit Care Med.

[16] Motulsky HJ, Ransnas LA (1987). Fitting curves to data using nonlinear regression: a practical
and nonmathematical review. FASEB J.

[17] Rossiter HB, Ward SA, Doyle VL, Howe FA, Griffiths JR, Whipp BJ (1999). Inferences from pulmonary O_2_ uptake with respect to
intramuscular [phosphocreatine] kinetics during moderate exercise in
humans. J Physiol.

[18] van Gestel AJR, Baty F, Rausch-Osthof AK, Brutsche MH (2014). Cardiopulmonary and gas-exchange responses during the six-minute
walk test in patients with chronic obstructive pulmonary
disease. Respiration.

[19] Kern L, Condrau S, Baty F, Wiegand J, van Gestel AJR, Azzola A (2014). Oxygen kinetics during 6-minute walk tests in patients with
cardiovascular and pulmonary disease. BMC Pulm Med.

[20] Sullivan GM, Feinn R (2012). Using effect size or why the P value is not
enough. J Grad Med Educ.

[21] Yazdanyar A, Aziz MM, Enright PL, Edmundowicz D, Boudreau R, Sutton-Tyrel K (2014). Association between 6-minute walk test and all-cause mortality,
coronary heart disease-specific mortality, and incident coronary heart
disease. J Aging Health.

[22] Tallaj JA, Sanderson B, Breland J, Adams C, Schumann C, Bittner V (2001). Assessment of functional outcomes using the 6-minute walk test in
cardiac rehabilitation: comparison of patients with and without left
ventricular dysfunction. J Cardiopulm Rehabil.

[23] Kervio G, Ville NS, Leclercq C, Daubert JC, Carre F (2004). Cardiorespiratory adaptations during the six-minute walk test in
chronic heart failure patients. Eur J Cardiovasc Prev Rehabil.

[24] Rocco IS, Viceconte M, Pauletti HO, Matos-Garcia BC, Marcondi NO, Bublitz C (2017). Oxygen uptake on-kinetics during six-minute walk test predicts
short-term outcomes after off-pump coronary artery bypass
surgery. Disabil Rehabil.

[25] Beatty AL, Schiller NB, Whooley MA (2002). Six-minute walk test as a prognostic tool in stable coronary
heart disease: data from the heart and soul study. Arch Intern Med.

[26] Radford MJ, Arnold JM, Bennett SJ, Cinquegrani MP, Cleland JGF, Havranek EP (2005). ACC/AHA key data elements and definitions for measuring the
clinical management and outcomes of patients with chronic heart failure: a
report of the American College of Cardiology/American Heart Association Task
Force on Clinical Data Standards (Writing Committee to Develop Heart Failure
Clinical Data Standards). Circulation.

[27] Harris KM, Anderson DR, Landers JD, Emery CF (2017). Utility of walk tests in evaluating functional status among
participants in an outpatient cardiac rehabilitation program. J Cardiopulm Rehabil Prev.

[B28] Sutherland N, Harrison A, Doherty P (2018). Factors influencing change in walking ability in patients with
heart failure undergoing exercise-based cardiac
rehabilitation. Int J Cardiol.

[29] Mazzoni G, Sassone B, Pasanisi G, Myers J, Mandini S, Volpato S (2018). A moderate 500-m treadmill walk for estimating peak oxygen uptake
in men with NYHA class I-II heart failure and reduced left ventricular
ejection fraction. BMC Cardiovasc Disord.

