# Supplementary material for: The body weight-walking distance product as a superior parameter in determining the VO2 on-kinetics in coronary artery disease
Source: Braz J Med Biol Res. 2025 May 30;58:e14367. doi: 10.1590/1414-431X2025e14367 (PMC12128776; doi:10.1590/1414-431X2025e14367)
Supplement: Supplementary file 1 [file 1414-431X-bjmbr-58-e14367-suppl.pdf]

Table S1. Gas-exchange parameters and cardiopulmonary responses during the 6-minute walk test.

	Rest	Steady state	P-value
HR, bpm	64.29 (10.89)	90.35 (19.72)	<0.0001
VO_2, mL/min	266.5 (52.4)	855.8 (252.4)	<0.0001
VO_2, $mL \cdot min^{-1} \cdot kg^{-1}$	3.81 (0.71)	12.22 (2.94)	<0.0001
VCO_2, mL/min	235.6 (48.3)	822.7 (258.7)	<0.0001
RER	0.88 (0.10)	0.96 (0.09)	0.0015
METS	1.09 (0.20)	3.49 (0.84)	<0.0001
VE, L/min	9.88 (2.49)	31.77 (9.67)	<0.0001
VE/VO_2	37.06 (7.08)	37.12 (6.43)	0.965
VE/VCO_2	41.94 (5.57)	38.61 (5.20)	<0.001
BR, %	85.07 (6.90)	56.63 (20.74)	<0.0001
RPE, dyspnea	0.2 (0.6)	2.6 (1.3)	<0.0001
RPE, limb discomfort	0.1 (0.4)	2.4 (1.4)	<0.0001

Data are reported as means and SD. Student's *t*-test. HR: heart rate; VO_2: oxygen uptake; VCO_2: carbon dioxide output; RER: respiratory exchange ratio; METS: metabolic equivalents; VE: ventilatory volume; VE/VO_2: ventilatory equivalent of oxygen; VE/VCO_2: ventilatory equivalent of carbon dioxide; BR: breathing reserve; RPE: Borg rating of perceived exertion scale.
